# Metagenomic Analyses of Plant Growth-Promoting and Carbon-Cycling Genes in Maize Rhizosphere Soils with Distinct Land-Use and Management Histories

**DOI:** 10.3390/genes12091431

**Published:** 2021-09-17

**Authors:** Chinenyenwa Fortune Chukwuneme, Ayansina Segun Ayangbenro, Olubukola Oluranti Babalola

**Affiliations:** Food Security and Safety Niche Area, Faculty of Natural and Agricultural Sciences, North-West University, Private Bag, X2046, Mmabatho 2735, South Africa; fortunechukwuneme@gmail.com (C.F.C.); 28072693@nwu.ac.za (A.S.A.)

**Keywords:** agricultural management practices, biogeochemical processes, crop productivity, nutrient mobilization, soil ecosystem functioning, soil fertility

## Abstract

Many studies have shown that the maize rhizosphere comprises several plant growth-promoting microbes, but there is little or no study on the effects of land-use and management histories on microbial functional gene diversity in the maize rhizosphere soils in Africa. Analyzing microbial genes in the rhizosphere of plants, especially those associated with plant growth promotion and carbon cycling, is important for improving soil fertility and crop productivity. Here, we provide a comparative analysis of microbial genes present in the rhizosphere samples of two maize fields with different agricultural histories using shotgun metagenomics. Genes involved in the nutrient mobilization, including *nif*A, *fix*J, *nor*B, *pst*A, *kef*A and B, and *ktr*B were significantly more abundant (α = 0.05) in former grassland (F1) rhizosphere soils. Among the carbon-cycling genes, the abundance of 12 genes, including all those involved in the degradation of methane were more significant (α = 0.05) in the F1 soils, whereas only five genes were significantly more abundant in the F2 soils. α-diversity indices were different across the samples and significant differences were observed in the β diversity of plant growth-promoting and carbon-cycling genes between the fields (ANOSIM, *p* = 0.01 and *R* = 0.52). Nitrate-nitrogen (N-NO_3_) was the most influential physicochemical parameter (*p =* 0.05 and contribution = 31.3%) that affected the distribution of the functional genes across the samples. The results indicate that land-use and management histories impact the composition and diversity of plant growth-promoting and carbon-cycling genes in the plant rhizosphere. The study widens our understanding of the effects of anthropogenic activities on plant health and major biogeochemical processes in soils.

## 1. Introduction

The plant rhizomicrobiome, comprising different microbial communities, plays immense roles in many processes of ecosystem functioning, such as nutrient recycling, suppressing disease pathogens, secreting plant growth-promoting enzymes, and mineralization of organic matter, which ultimately lead to increased soil fertility and crop productivity [1,2]. The rich nutrients provided by plants attract several microbes around the roots, which are recruited from the surrounding soils [3,4] by the metabolic compatibility and signaling events of exudates secreted by the plant roots into the rhizosphere environment, the host-plant immune system, and interactions among different microbes within the plant rhizosphere [5,6].

Soil ecosystem functioning is mostly determined by the activity and complexity of the inhabiting microbes. These microbes are influenced by several biological, chemical, and physical properties of the soil environment [1,7]. The microbial community composition in soils can be altered by different land-use and management practices, which consequently affect certain ecosystem functioning in soils [8,9]. Agricultural management practices impact microbial community structure and functions, thereby complexing the contributions of microbial communities towards important ecosystem functions such as nutrient and carbon cycling [10,11]. Most studies on the effects of land-use practices on the soil ecosystem functions focused mainly on the composition and structure of soil microbial communities [12,13]. Microbial community composition in different ecosystems and its effects on ecosystem functioning has been studied [14,15]. The literature has shown that the information obtained from the taxonomic composition and abundance of soil microbial communities alone is insufficient to predict their functional potentials [16,17]. Therefore, quantifying the knowledge of the functional capabilities of microbial communities in soils will help identify their roles in the ecosystems, how they are impacted by land-use and management practices, and their influence on soil ecosystem functions. such as organic matter mineralization, nutrient cycling, degradation of organic pollutants, and plant–microbe interactions [9,18,19].

Recent studies have suggested that the functions of soil microbial communities can be better predicted by studying their functional genes [20,21,22,23]. Knowledge of microbial functional gene composition and diversity in the rhizosphere of agricultural soils, instead of mere taxonomic insight, is important for understanding the dynamics of vital processes, such as nutrient and carbon cycling, and how they are influenced by agricultural management practices [23]. The whole metagenomic sequencing of rhizosphere soil samples has been tested to provide information on the diversity of microbial functional genes in the plant rhizosphere [24,25]. Metagenomics provides information on a wide variety of functional genes present in a sample, which may assist in the acquisition of information on the functional potentials of microbial communities in soils. Through this method, information on functional genes that play important roles in the rhizosphere soil ecosystem is obtained [26]. These functional genes may include specific ones, such as the phloroglucinol (*phl*) synthesis and nitrogen fixation (*nif*) genes that can directly confer beneficial traits to plants, and the pyrroloquinoline quinone (*pqq*) biosynthesis genes, which contribute to many indirect functions in plant cells. Together, these genes enhance plant growth and health [27]. 

Studies have reported the influence of land-use practices on the diversity of functional genes in agricultural soils [1,28]. However, studies on microbial functional gene diversity in the rhizosphere of plants in African soils as influenced by their land-use and management histories are rare. Moreover, the specific functional genes responsible for performing various important functions, such as the biogeochemical cycling of nutrients and plant-growth promotion in the maize rhizosphere, are understudied. This study provides a principal report on the effects of land-use history (conversion from grassland to cultivated land) and management practices (tillage and no tillage) on the diversity of plant growth-promoting and carbon-cycling genes in maize rhizospheric soils. 

To gain deeper knowledge on the diversity of genes concerned with plant growth promotion and carbon cycling, we analyzed the metagenomes of rhizospheric soil samples from former grassland soil and intensively cultivated agricultural land using shotgun metagenomic sequencing. We compared the composition and diversity of functional genes involved in plant growth promotion and carbon cycling in the rhizospheric soils of the former grassland with those from the intensively cultivated land. Furthermore, we compared the taxonomic profiles of one field with the other. Based on our previous study, we formulated two hypotheses. First, we assumed that although the maize rhizosphere harbored important beneficial microbes, this environment must also be characterized by diverse microbial functional genes that contribute to important functions in the soil ecosystem. Second, we expected the diversity of the genes that contribute to plant growth and carbon cycling in the two fields to differ significantly from one another due to their land-use and management histories.

## 2. Materials and Methods

### 2.1. Soil Sampling and Sites Description

Soil samples were collected in March 2019 from two maize plantations in Ventersdorp (F1) and Mafikeng (F2), South Africa (located in the semi-arid regions of the North West province). The choice of our sample collection was influenced by the agricultural histories of the two plantations. The field at Ventersdorp (approximately 130 hectares) has existed since 1962, initially as grassland for animal grazing, and subsequently converted to cultivable land in 2015, with no tilling system and fertilizer application (N = 90, P = 60, and K = 60 kg ha^−1^). On the other hand, the Mafikeng field has existed since 1989 as a continuously cultivated land, with a mechanized tilling system and fertilizer applied at the rates of N = 140, P = 80, and K = 80 kg ha^−1^. The average winter and summer temperatures at Ventersdorp are 10.6 and 22.9 °C, respectively, with an annual average rainfall of 585 mm, an average precipitation of 4 mm (in winter) and 105 mm (in summer), an altitude of 1339 m, and average humidity of 47% (in winter) and 45% (in summer). On the other hand, the mean temperatures in Mafikeng are 11 and 23.1 °C in winter and summer, respectively, with an annual rainfall of 571 mm, average precipitation of 3 mm in winter and 96 mm in summer, an altitude of 1290 m, and mean humidity of 46% and 44% in winter and summer, respectively. Soil samples were collected from the following locations: 26°19′38″ S and 26°53′18″ E for F1, and 25°48′00″ S and 25°38′21″ E for F2. Rhizosphere soils were collected by deliberately uprooting maize plants and agitating the uprooted plant to remove loosely attached soils; meanwhile, soils attached to the root crowns, where rooting was so dense, were collected together with the roots in sterile plastic bags. Before sample collection, each field was split into four parts (representing north, south, east, and west), and each part was called a site. In each site, rhizospheric soil samples were collected from six different maize plants, later pooled to form a composite sample. Therefore, for F1, rhizospheric soil samples were collected from sites GZ1, GZ2, GZ3, and GZ4, and for F2, we collected samples from sites AG1, AG2, AG3, and AG4. A total of 8 composite soil samples were collected from the rhizosphere of maize plants in the two fields (4 from each field). Samples were collected at the flowering stage, and soils from both fields were typical of loamy sand. These samples were stored in a cooler containing ice and transported to the laboratory. At the laboratory, each sample was divided into two, one for physicochemical analysis and the other for whole DNA extraction. Samples were kept at −20 °C until they were needed for analyses.

### 2.2. Physicochemical Analyses of Soil Samples

The chemical properties of soil samples were determined using 20 g of dried and sieved soils that were kept specifically for this purpose. The pH of samples was determined using a pH meter at a soil-to-water ratio of 1:2.5. The Kjeldahl method was used to determine the amount of nitrogen in the samples [29], while the procedure of Bray and Kurtz [30] was employed to determine the extractable phosphorus content of the samples. Organic carbon (OC) content was measured using the method of Walkley and Black [31] and Shi et al. [32]. Exchangeable potassium, N-NO_3_, and N-NH_4_ content, and the available sulfur in samples were determined after extraction with 1 M ammonium acetate, 2 M potassium chloride (KCl), and 0.1 M hydrochloric acid (HCl), respectively, while the amount of organic matter was estimated by the loss-on-ignition (LOI) method [33].

### 2.3. Metagenomic DNA Extraction and Sequencing

A DNeasy PowerMax Soil Kit (Qiagen, Germantown, MD, USA) was used to extract whole microbial DNA from 5 g of soil samples according to the manufacturer’s guidelines. The initial DNA concentration of the samples was confirmed using Qubit^®^ dsDNA HS Assay Kit (Life Technologies, Carlsbad, CA, USA), and samples were subjected to further cleaning using the DNEasy PowerClean Pro Cleanup Kit (Qiagen, Germantown, MD, USA). Whole metagenome sequencing of samples through the shotgun approach, which generated the complete datasets for the study, was performed at the Molecular Research Laboratories (MR DNA, Shallowater, TX, USA). Adhering to user instructions step-by-step, metagenomic DNA libraries were developed with 20–25 ng of DNA using a Nextera DNA Flex library preparation kit (Illumina, San Diego, CA, USA). After sample cleanup, the quality of the DNA in samples was again checked using the Qubit^®^ dsDNA HS Assay Kit (Life Technologies, Carlsbad, CA, USA). The quality DNA samples were made to undergo simultaneous fragmentation with the subsequent addition of adapter sequences. A limited-cycle PCR was run on the samples and unique indices were added. The Qubit^®^ dsDNA HS Assay Kit (Life Technologies, Carlsbad, CA, USA) was reused to check the final library concentration, while the Agilent 2100 Bioanalyzer (Agilent Technologies, Santa Clara, CA, USA) was used to deduce the average size of the developed libraries. The libraries were pooled and diluted to 0.6 nM. Afterward, 300-cycle pair-end sequencing was performed on the libraries using the NovaSeq system on Illumina. 

### 2.4. Metagenome Sequence Processing and Analysis of Data

The raw sequences of each metagenome were uploaded to the metagenomics rapid annotation pipeline (MG-RAST) version 4.0.3 server [34], and quality control was performed on the sequences. The preprocessing steps (quality control and trimming) involved the elimination of artificial sequences, which were products of sequence artifacts, the removal of sequences with over 5 ambiguous base pairs (bp) and a 15 phred cutoff score, removal of host species-specific sequences, and the removal of sequences whose mean lengths were more significant than 2 standard deviations from the mean. Afterward, sequences were annotated using the BLAST-like alignment (BLAT) algorithm [35] on the M5NR database, which provides a nonredundant combination of different databases [36]. Taxonomic groupings were performed using the Ribosomal Database Project (RDP), while the assignment of protein-coding genes (at the level of functions) were performed on the SEED subsystems database. The SEED subsystems database on the MG-RAST server is a continuously updated genome database, application programming interface (API), web front end, and server scripts consisting of integrated genomic data and collections of functionally related protein families that are used to predict gene functions and new pathways [37]. BlastX was used to detect hits that have maximum lengths of 15 bp, an *E*-value cutoff of 1 × 10^−5^, and a 60% minimum identity. Sequences with failed annotation did not receive any further analysis. We were interested in the entire microbiome comprising archaea, bacteria, and fungi. However, data from protists and other micro-eukaryotes were excluded from the analysis. The effect of experimental noise or error was minimized by applying the “normalized data” option on the MG-RAST server. We manually selected and arranged the genes imparting plant-beneficial functions from the data we obtained for the microbial functions in the SEED subsystems database. The 8 sequences of this study were individually evaluated on the MG-RAST server, and data analyses were performed on all the metagenome samples (GZ1–GZ4 and AG1–AG4). The metagenomic sequences of the study samples are available in the NCBI SRA dataset, with the BioProject accession number PRJNA649682.

Shannon, Evenness, and Simpsons were used as parameters to determine the α diversity indices of all the samples. These indices were used to compare habitats using the Kruskal–Wallis test. Significantly abundant microbial genes were identified as biomarkers of the microbial communities, and the samples were determined using the linear discriminant analysis (LDA) effect size (LEfSe) version 1.0. Here, the logarithmic LDA cutoff score was set to 2.0, and the α parameter significance threshold for the Kruskal–Wallis test performed among classes was set to 0.05. To determine the β diversity of the plant growth-promoting and carbon-cycling genes, we used the principal coordinate analysis (PCoA) centered on the Euclidean distance matrix. To identify significantly different metagenomes among the samples, we used the one-way analysis of similarities (ANOSIM) through 9999 permutations. The percentage (%) contribution of each functional gene to the dissimilarities in gene abundance between and within the sample groups was assessed using the similarity percentage (SIMPER) analysis. The canonical correspondence analysis (CCA), through the forward selection option, was used to determine the physicochemical parameters that best described the functional genes, while the Monte Carlo permutation test with 9999 random permutations was used to measure the significance. All the physicochemical parameters of the soils were incorporated into the CCA analysis as descriptive variables.

**Figure 1:**
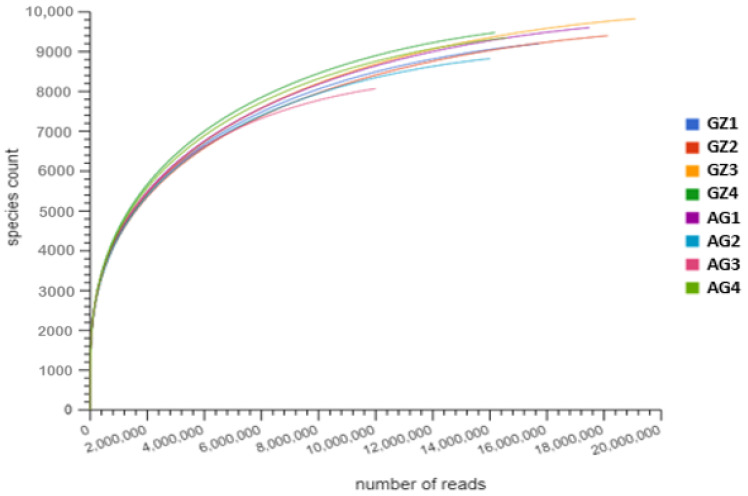
Rarefaction curve depicting sample richness and the sample sites from which rhizospheric samples were collected.

The rarefaction curve was obtained after the normalization of the datasets using the analysis tools in MG-RAST v. 4.0.3 [34] (Figure 1). Heatmaps showing the abundances of microbial families, plant growth-promoting and carbon-cycling genes, and an extended error bar plot used to identify the significant microbial communities in the samples, were obtained using the statistical analysis of taxonomic and functional profiles (STAMP), version 2.1.3 [38]. The α diversity, ANOSIM, and SIMPER analyses were performed using the PAST version 3.20 software, developed by Hammer et al. [39], while the PCoA and CCA were performed in CANOCO 5 (Microcomputer Power, Ithaca, 148 NY, USA). The Galaxy software on https://huttenhower.sph.harvard.edu/galaxy/ (accessed December 2020) was used to plot the bar chart showing the statistically differential and biologically consistent differences in the abundance of functional genes involved in plant growth promotion and carbon cycling in the samples [40].

## 3. Results

### 3.1. Soil Chemical Analysis

The results of the soil chemical analyses of this study have been previously described in Chukwuneme et al. [41]. Briefly, the pH of the GZ samples was between 6.45 and 7.04 (neutral), while that of the AG samples ranged between 4.84 and 5.49 (acidic). The sulfur content of both soils was low and observed only in the GZ1, GZ3, and AG2 samples, while the phosphorus contents of the GZ and AG samples ranged from 19.75 (GZ2) to 40.39 (GZ3) mg/kg and from 16.25 (AG4) to 56.88 (AG2) mg/kg, respectively. Moreover, total C, organic C, organic matter, and nitrate-nitrogen (NO_3_-N) contents were higher in the GZ samples compared to the ammonium (N-NH_4_) content, which was higher in the AG samples (Table S1).

### 3.2. Assembly and Analysis of Shotgun Metagenomic Sequence Data

From the rarefaction curve in Figure 1, most of the sample reads reached saturation points, thereby indicating a full coverage of the sampling efforts. After quality control on the MG-RAST server, the output file obtained contained sequences in the range of 5,255,550–9,039,015 and 2,627,486–8,287,108 for the GZ1–GZ4 and the AG1–AG4 rhizosphere samples, respectively. Out of the quality sequences obtained, 39.63–40.98% of the sequences in the GZ samples contained proteins whose functions are known, while for the AG samples, the proteins with known functions ranged from 41.39 to 43.42%. Furthermore, the quality sequences obtained contained 2,732,830–4,759,994 and 1,306,964–4,262,335 proteins for the GZ (GZ1–GZ4) and AG (AG1–AG4) samples, respectively, with unknown functions.

### 3.3. The Distribution of Microbes across the Maize Rhizosphere Soils

**Figure 2:**
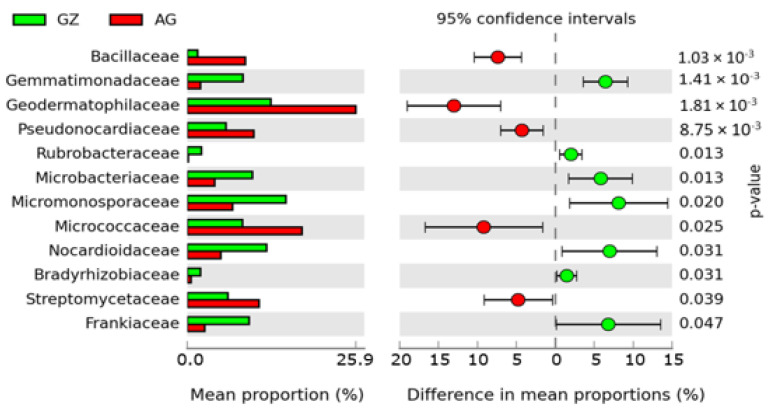
An extended error bar plot showing the significantly abundant microbial families in the former grassland (GZ) and the intensively cultivated (AG) soils.

Metagenomic analysis using the RDP database revealed the dominance of 14 bacterial families as the most abundant microbial families observed in the former grassland and the intensively cultivated soils (Figure S1). Among these families, the abundance of eight bacterial families, including Micromonosporaceae (GZ1), Nocardioidaceae (GZ4), Gemmatimonadaceae (GZ1), Microbacteriaceae (GZ3), Frankiaceae (GZ3), and others were highest in the F1 soils, while the abundance of six bacterial families, including Geodermatophilaceae (AG3), Pseudonocardiaceae (AG4), Micrococcaceae (AG2), and others was predominant in the F2 soils (Figure S1). Among the archaeal families, Methanobacteriaceae was found in GZ3, AG1, and AG2, Methanomicrobiaceae in GZ2 and GZ4, and Thermofilaceae in the AG2 samples, whereas Ustilaginaceae (the only fungi found) was observed in all samples except AG2 and AG3. We used the STAMP software to identify significantly abundant microbial communities in the samples, represented by the extended error bar plot (Figure 2). The analysis showed that out of 18 microbial families, seven of them were significantly more abundant (*p* < 0.05) in the F1 samples, whereas five others were significantly more abundant (*p* < 0.05) in the F2 rhizosphere (Figure 2). However, no significant difference (Kruskal–Wallis, *p* = 0.98) was observed in the α diversity of the microbial communities between the F1 and the F2 soils.

### 3.4. Microbial Genes That Enhance Plant Growth and Fitness Observed in the Maize Fields 

The SEED subsystem database used in the functional classification of the metagenomic dataset revealed the abundance of several genes involved in plant growth promotion and carbon cycling in the agricultural soils (Tables S2 and S3).

#### 3.4.1. Genes That Facilitate Nutrient Mobilization and Plant Growth

**Figure 3:**
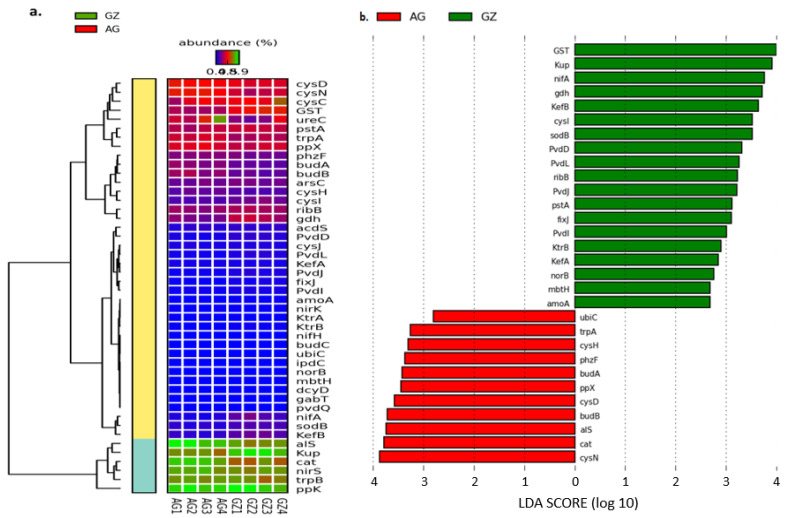
(**a**) Heatmap representing the composition of plant growth-promoting genes in maize rhizosphere samples and (**b**) a bar plot of linear discriminant analysis (LDA) scores showing the differentially abundant plant growth-promoting genes in the rhizosphere samples. The vertical axis (Figure 3b) represents the plant growth-promoting genes whose differences between the sample groups were significant, while the horizontal axis depicts the LDA, showing the LDA score (log 10) of the corresponding plant growth-promoting genes. GZ and AG stand for rhizosphere samples from the former grassland and the intensively cultivated soils, respectively.

The metagenomic analysis of microbial functional genes in our samples using the SEED subsystems revealed a total of 8091 functional genes, of which several involved in the cycling of major nutrients (nitrogen, phosphorus, and potassium) that promote soil fertility were identified (Table S2). The plant growth-promoting genes assessed in our study were those involved in nitrogen fixation (*nif*A, *nif*H, and *fix*J), nitrification (*amo*A), denitrification (*nir*K, *nir*S, and *nor*B), phosphorus cycling (*gdh*, *pp*X, *pp*K, and *pst*A,), potassium cycling (*kef*A, *kef*B, *kup*, *ktr*A, and *ktr*B), ACC deaminase activity (*acd*S and *dcy*D), IAA biosynthesis (*ipd*C), tryptophan biosynthesis (*trp*A and *trp*B), sulfur metabolism (*cys*C, *cys*D, *cys*H, *cys*I, *cys*J, and *cys*N), pyoverdine siderophore biosynthesis (*pvd*D, *pvd*I, *pvd*J, *pvd*L, *pvd*Q, and *mbt*H), acetoin and 2,3-butanediol biosynthesis (*al*S, *bud*A, *bud*B, and *bud*C), phenazine biosynthesis (*phz*F), oxidative stress (*GST*, *sod*B, and *cat*), quorum sensing (*rib*B), arsenate and atrazine degradation (*ars*C, *gab*T, and *ure*C), and 4-hydroxybenzoate biosynthesis (*ubi*C) (Table S2). The highest abundances of the genes involved in nitrogen cycling—*nif*A, *fix*J, *amo*A, *nir*K, *nir*S, and *nor*B—were observed in the F1 rhizosphere (GZ2, GZ3, GZ2, GZ3, GZ3, and GZ3, respectively) (Figure 3a and Table S2). Notably, the highest abundances of the genes concerned with phosphorus cycling, including glucose dehydrogenase (*gdh*), polyphosphate kinase (*ppk*), exopolyphosphatase (PPX), and the phosphate transport system (*pst*A) were observed in GZ3. Among the genes involved in potassium cycling, we observed the highest abundances of the potassium efflux (*kef*A and B) and the potassium uptake (*kup*) genes in GZ3, while the highest abundance of the potassium transport (*ktr*A) gene was observed in AG1 (Figure 3a and Table S2). 

The analysis also revealed the abundance of various genes involved in both the direct and indirect enhancement of plant growth and health. The abundances of the *ipd*C, *trp*A, *trp*B, *bud*A, *bud*B, and *al*S genes were highest in AG1 compared to *dcy*D, *bud*C, *phz*F, *ars*C, *sod*B, and *GST*, whose highest abundances were observed in GZ3 (Figure 3a and Table S2). Further analysis of genes involved in plant growth promotion using the linear discriminant analysis (LDA) effect size (LEfSe) on the galaxy server revealed the differences in the composition of these genes between the fields by describing their effect sizes. While performing the analysis, we used the strict (all classes differential) version, which identified 19 of the 46 plant growth-promoting genes, showing statistically differential and biologically consistent differences (α = 0.05) in the GZ samples (Figure 3b). In the AG samples, 11 plant growth-promoting genes with statistically differential abundance were observed (Figure 3b). The results revealed that the genes *GST* and *kup* (with an LDA score nearly 4 orders of magnitude) were the most differentially abundant (α = 0.05) plant growth-promoting genes observed in the GZ samples, whereas the *cys*N and *cat* (with an LDA score over 3.5 orders of magnitude) were the most differentially abundant (α = 0.05) in the AG samples (Figure 3b). The differences observed in the abundance of these genes among the samples reached a threshold of 2.75 for the GZ samples and 2.85 for the AG samples (Figure 3b).

**Figure 4:**
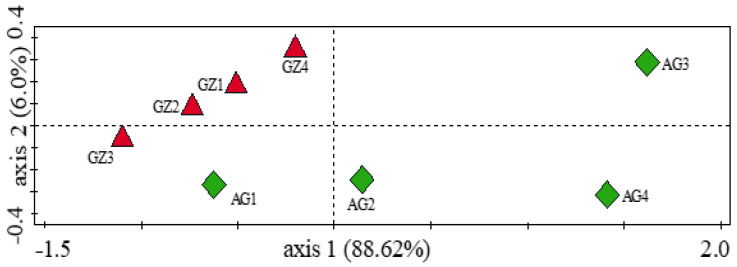
Principal coordinate analysis (PCoA) of genes involved in plant growth promotion found in maize rhizosphere samples.

To evaluate the α diversity of plant growth-promoting genes in the samples, we used the Simpson, Shannon, and Evenness indices. The α diversity analysis indicated that there was no significant difference (Kruskal–Wallis, *p* = 0.94) in the diversity of genes involved in plant growth promotion between the samples. From the α diversity analyses, the Simpson index was the same (0.95) in all samples. However, the Shannon and Evenness indices were higher in the GZ than in the AG samples (Figure S2). The β diversity (the diversity between the GZ and AG samples), which was determined using the analysis of similarity (ANOSIM), revealed that there was a significant difference (*p* = 0.01 and *R* = 0.52) in the diversity of these genes among the samples, thereby confirming the result obtained from the principal coordinate analysis (PCoA), which displayed a distinct separation between the GZ and AG samples and a close clustering of the GZ samples (Figure 4). The similarity percentage (SIMPER) analysis showed an overall average dissimilarity of 28 in the plant growth-promoting genes between all GZ and AG samples, with the *kup* gene contributing the most (8.70%) to the dissimilarity (Table 1). On the other hand, within the GZ samples (GZ1–GZ4), we observed the highest overall average dissimilarity of 24.20 between the GZ3 and GZ4 samples, whereas among the AG samples (AG1–AG4), the highest overall average dissimilarity of 48.0 was observed between the sample pair AG1 and AG3 (Table 1). 

#### 3.4.2. Microbial Genes Involved in Carbon Cycling

**Figure 5:**
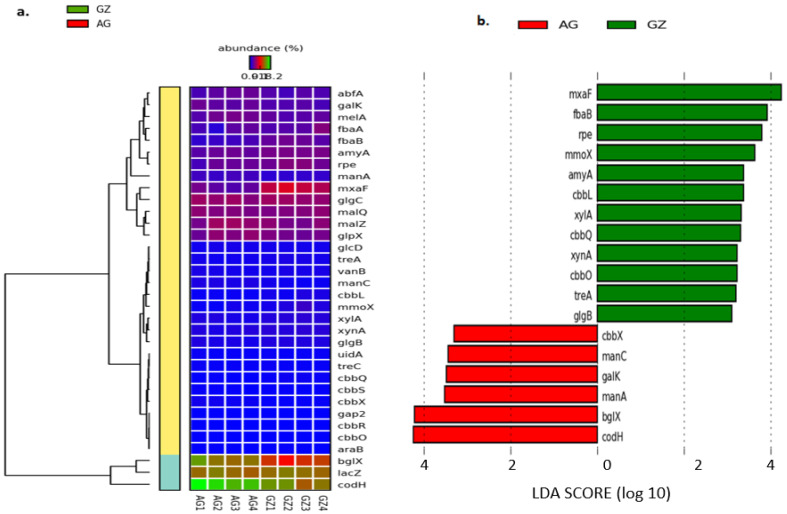
(**a**) Heatmap representing the composition of carbon-cycling genes in maize rhizosphere samples and (**b**) a bar plot of linear discriminant analysis (LDA) scores showing the differentially abundant carbon-cycling genes in the rhizosphere samples. The vertical axis (Figure 5b) represents the carbon-cycling genes whose differences between the sample groups were significant, while the horizontal axis depicts the LDA, showing the LDA score (log 10) of the corresponding carbon cycling gene. GZ and AG stand for rhizosphere samples from the former grassland and the intensively cultivated soils, respectively.

Genes that were considered to be associated with carbon cycling were identified using the functional category of the SEED subsystems (Table S3). From our analysis of the metagenomes in the maize rhizosphere soils, we found 34 important genes that were linked with carbon cycling in all samples. These genes include those concerned with the metabolism of carbohydrate (*gal*K, *glc*D, *man*A, *man*C, *mel*A, and *lac*Z), the fixing of carbon (*cod*H, *cbb*L, *cbb*R, *cbb*O, *cbb*Q, *cbb*X, *cbb*S, *gap*2, and *rpe*), and the degrading of starch (*amy*A, *glg*B, *glg*C, *bgl*X, *mal*Z, *mal*Q, *abf*A, *tre*A, and *tre*C), methane (*glp*X, *fba*A, *fba*B, *mxa*F, and *mmo*X), hemicellulose (*ara*B, *xyl*A, and *xyn*A), and xenobiotics (*van*B and *uid*A). With respect to carbon fixation, the abundance of the *cbb*R, *cbb*S, *cbb*X, and *cod*H genes were highest in the AG rhizosphere compared to the *gap*2, *rpe*, *cbb*L, *cbb*O, and *cbb*Q genes whose abundances were observed to be highest in the GZ rhizosphere. Interestingly, all the methane-degrading genes (*mxa*F, *mmo*X, *fba*A, *fba*B, and *glp*X) were more abundant in the GZ than in the AG rhizosphere (Figure 5a and Table S3). The abundance of several genes involved in starch degradation, including *amy*A, *glg*B, *glg*C, *mal*Z, *mal*Q, *abf*A, and *tre*A, was highest in the GZ samples. Furthermore, the highest abundances of several genes involved in hemicellulose degradation (*ara*B, *xyl*A, and *xyn*A), carbohydrate metabolism (*lac*Z, *mel*A, and *glc*D), and xenobiotics degradation (*van*B) were observed in the GZ samples (Figure 5a and Table S3). By employing the strict (all classes differential) version of the linear discriminant analysis (LDA) effect size (LEfSe), we determined the effect sizes of differences in the abundance and distribution of carbon-cycling genes between the fields (GZ = GZ1–GZ4 and AG = AG1–AG4). The output revealed 12 and 6 carbon-cycling genes (out of the 34 genes), showing statistically differential and biologically consistent differences in the GZ and AG samples (Figure 5b). From the analysis, the most differentially abundant (α = 0.05) carbon-cycling genes in GZ were those concerned with methane degradation (*mxa*F, *fba*B, *rpe*, and *mmo*X), with high LDA scores (over 3 orders of magnitude), reaching a threshold of 4.3 (Figure 5b). On the other hand, the most differentially abundant (α = 0.05) carbon-cycling genes observed in the AG samples were the *cod*H and *bgl*X genes, involved in carbon fixation and starch degradation, with high LDA scores of 4.3 (Figure 5b).

**Figure 6:**
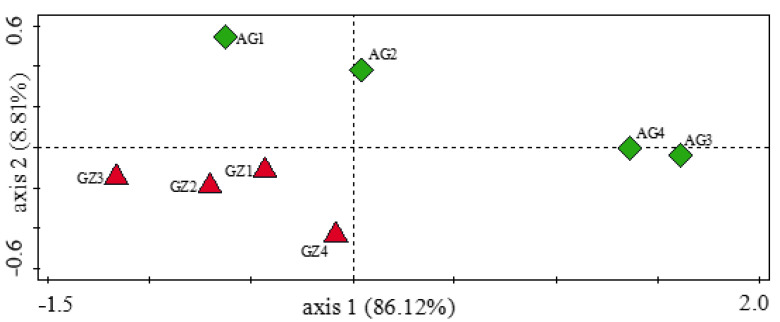
Principal coordinate analysis (PCoA) of genes involved in carbon cycling found in the maize rhizosphere samples.

To determine the α diversity of carbon-cycling genes in the rhizosphere samples, we used the Simpson, Shannon, and Evenness indices. The values obtained from these indices were higher in the GZ samples than in the AG samples (Figure S3). However, no significant difference (Kruskal–Wallis, *p* = 0.93) was observed in the α diversity of the carbon-cycling genes between the fields. On the other hand, ANOSIM, which was used to determine the β diversity, indicated that there was a significant difference (*p* = 0.01 and *R* = 0.52) in the β diversity of the carbon-cycling genes between the samples from the former grassland and the intensively cultivated land. This analysis is a confirmation of the principal coordinate analysis (PCoA), which showed a distinct separation of the samples from the former grassland and the intensively cultivated land (Figure 6). From the SIMPER analysis, an overall pairwise dissimilarity of 26 was observed in the diversity of carbon-cycling genes between the GZ and the AG samples (Table 1). The diversity of carbon-cycling genes within the GZ samples showed that the highest pairwise dissimilarity of 24.15 was observed between the GZ3 and GZ4 samples compared to 45.58 (between AG1 and AG3) obtained as the highest pairwise dissimilarity observed in the AG samples. The top shared carbon-cycling genes that contributed the most to the observed differences in the sample groups and their percentage contributions are presented in Table 1.

### 3.5. Influence of Soil Physicochemical Properties on the Diversity of Carbon-Cycling and Plant Growth-Promoting Genes

**Figure 7:**
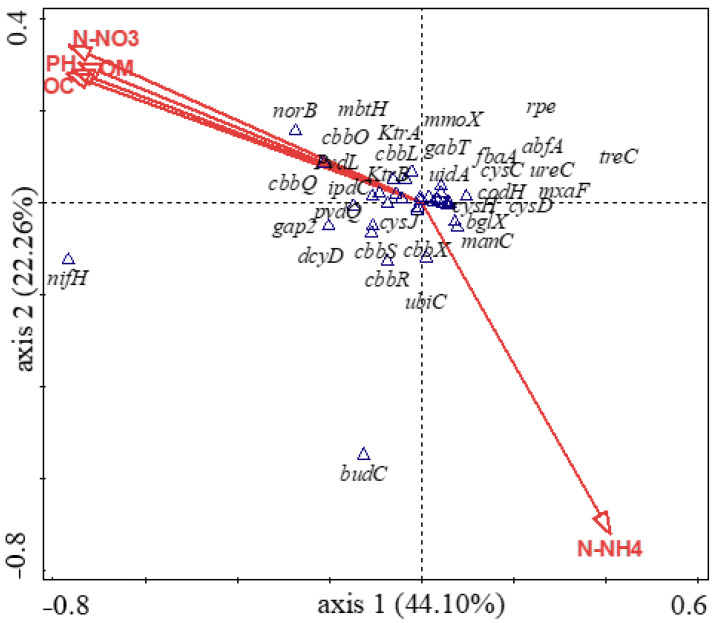
Canonical correspondence analysis (CCA) showing the effect of the soil physicochemical analysis on the diversity and composition of the genes involved in carbon cycling and plant growth promotion across the samples. Legend: N-NO_3_ represents nitrate-nitrogen, N-NH_4_ represents ammonium nitrogen, OC = organic carbon, and OM = organic carbon. Plant growth-promoting genes: *bud*C—acetoin (diacetyl) reductase (EC 1.1.1.5), *cys*C—adenylylsulfate kinase, *cys*D—sulfate adenylyltransferase subunit 2, *cys*H—phosphoadenylyl-sulfate reductase (thioredoxin), *cys*J—sulfite reductase (NADPH) flavoprotein α-component, *dcy*D—D-cysteine desulfhydrase, *gab*T- GABA aminotransferase, *ipd*C—indole-3-pyruvate decarboxylase, *ktr*A—potassium uptake protein A, *Ktr*B—potassium uptake protein B, *mbt*H—hypothetical MbtH-like protein, *nif*H—nitrogenase (molybdenum-iron) reductase and maturation, *nor*B—nitric oxide reductase subunit B, *pvd*L—pyoverdine chromophore precursor synthetase, *pvd*Q—acyl-homoserine lactone acylase, *ubi*C—chorismate-pyruvate lyase, and *ure*C—urease subunit α. Carbon-cycling genes: *abf*A—α-N-arabinofuranosidase, *bgl*X—β-glucosidase, *cbb*L—RuBisCo large chain, *cbb*O—RuBisCo activation protein, *cbb*Q—RuBisCo activation protein, *cbb*R—RuBisCo operon transcriptional regulator, *cbb*S—ribulose bisphosphate carboxylase small chain, *cbb*X—probable RuBisCo-expression protein, *cod*H—carbon monoxide dehydrogenase large chain, *fba*A—fructose-bisphosphate aldolase class I, *gap*2—NAD(P)-dependent glyceraldehyde 3-phosphate, *man*C—mannose-1-phosphate guanylyltransferase, *mmo*X—methane monooxygenase component A α chain, *mxa*F—methanol dehydrogenase large subunit protein, *rpe*—ribulose-phosphate 3-epimerase, *tre*C—trehalose-6-phosphate hydrolase, and *uid*A—β-glucuronidase.

In this study, we employed the canonical correspondence analysis (CCA) to assess the correlation between the microbial functional genes in the study and the soils’ physicochemical parameters. We computed the CCA plot with all the environmental variables, as presented in Figure 7, and observed a CCA permutation test of 0.015, indicating that the composition of the carbon-cycling and plant growth-promoting genes were affected by the physicochemical properties of the soils (Figure 7). A correlation analysis showed that *cys*C, *cys*J, *dcy*D, *ipd*C, *pvd*Q*, pvd*L, *cbb*L, *cbb*Q, *rpe*, *tre*C, *gap*2, *mxa*F, *mmo*X, and *uid*A negatively correlated with N-NH4 and positively correlated with N-NO_3_, N-NH_4_, pH, OC, and OM. On the other hand, *cbb*X, *ure*C, and *man*C positively correlated with N-NH_4_ and negatively correlated with N-NO_3_, N-NH_4_, pH, OC, and OM. However, *abf*A, *cbb*R, and *cbb*S positively correlated with all the tested variables (N-NO_3_, N-NH_4_, pH, OC, and OM). The forward selection option through the Monte Carlo permutation test, with 9999 random permutations, was used to study which soil chemical parameter was the most influential in the differences observed in the microbial functional genes across the samples (Table S4). From the analysis, we found that N-NO_3_ contributed the most (31.3%) to the variation, with a *p*-value of 0.05 (Table S4).

## 4. Discussion

The practice of land-use conversion has become common over the years, with studies reporting its impacts on soil microbial communities and the environment; however, the effects of land-use history on microbial functional gene potential in the rhizosphere of plants remain understudied. Using shotgun metagenomic sequencing, this study revealed the differences in microbial functional genes, particularly those involved in nutrient mobilization, plant growth promotion, and carbon cycling of land previously used as pasture (with a lower N fertilizer application rate and a no-tillage system), and of land that had been under intensive cultivation (with a higher N fertilizer application rate and a conventional tillage system) for several years. We showed that the abundance and diversity of the genes involved in plant growth-promotion and carbon cycling within these fields are distinct from one another, thereby representing the land-use and management histories of the fields. The soils also differed in their physicochemical properties, which also contributed to the differences in the diversity of the genes. This study highlighted the effects of land-use and management histories as well as soil chemical properties on the functioning and maintenance of the soil ecosystem, especially in nitrogen and carbon cycling, in order to present the long-term effects of cultivation on these functional genes in the plants’ rhizosphere. Understanding the long-term effects of land-use and management practices on the diversity of microbial functional genes helps to explain how the soil ecosystem copes with current and future agricultural management practices.

Although the rhizosphere metagenomes contained sequences that originate from the archaeal and fungal groups, sequences from the bacterial families were more predominant as they represented more than 99% of the sequences. The analysis of microbial families in the study revealed that some bacterial communities were more predominant in one field than the other, thereby indicating the possible effects of land use on microbial community composition and diversity. Moreover, our analysis revealed that the fields were dominated by important microbial communities known to improve plant growth and degrade complex polysaccharides, including chitin, lignin, cellulose, and hemicellulose, and plant residues in soils [42]. Micromonosporaceae, Microbacteriaceae, Nocardioidaceae, and Bradyrhizobiaceae (significantly more abundant in the F1 rhizosphere samples) are important plant colonizers and have been implicated in plant growth promotion in various agricultural soils [43]. Their contributions toward plant growth and development include the production, regulation, and degradation of phytohormones, the production of siderophores, mineralization and mobilization of soil nutrients, production of vitamins for plant growth, antagonism against various phytopathogens, among other important functions in the soil [43,44,45]. The abundance of these communities in the fields indicate their relevance in maintaining soil fertility and plant health in the soils. We also suggested that the differentially abundant microbial families (Figure 2) might likely be important, particularly in the functioning of the host plants, and contribute immensely towards the plants’ health and fitness as proposed by Hartman et al. [46] and Pérez-Jaramillo et al. [47].

The composition of microbial functional genes involved in plant growth promotion in the rhizosphere soils was significantly affected by the soils’ management regimes. The composition of genes involved in plant growth promotion in the former grassland (GZ) soils differed from those of the intensively cultivated land (AG). From the analyses, the higher abundance of genes involved in nitrogen cycling in the GZ (F1 rhizosphere) samples indicates that management practices impact the abundance of these genes in the plant rhizosphere. The differences observed in the abundance of genes concerned with nitrogen fixation (*nif*A and *fix*J) in the soils further suggest that reduced fertilizer application rates might increase biological nitrogen fixation in agricultural soils. Yu et al. [48] also reported a higher abundance of the nitrogen fixation gene, *nif*H, in a reduced fertilizer-treated soil. Our results on the abundance of nitrogen fixation genes is also evidenced by the higher abundance of the nitrogen-fixing microbial communities—the Micromonosporaceae, Frankiaceae, and Bradyrhizobiaceae families [49,50]—observed in GZ soils (Figure 2 and Figure S1). The data concurs with previous studies that suggest that the amount of biological nitrogen fixation in lower N-fertilized soils is higher compared to higher N-fertilized soils [48,51]. Moreover, land tilling also affects the abundance of genes involved in several stages of nitrogen fixation [52]. Under low N fertilization (67 kg ha^−1^), Hu et al. [53] reported that the relative abundance of the *nif*H transcript was higher in no-till soils than in conventionally tilled soils. Furthermore, the influence of soil management was also observed in the abundance of genes involved in nitrification (*amo*A) and denitrification (*nor*B, *nir*K, and *nir*S) in the soils. Hu, Jin, Konkel, Schaeffer, Schneider and DeBruyn [53] studied the effects of agricultural management on the abundance of genes involved in nitrogen cycling. They observed that under N fertilizer treatment (67 kg ha^−1^), both the relative abundance and transcript of the *amo*A gene was increased in no-till soils. However, for the denitrification genes, the relative abundance of *nir*K increased in no-till plots, whereas the *nir*S gene was higher in conventionally tilled plots. These results confirm that N fertilization promotes denitrification and nitrification in agricultural soils, and that reduced fertilization management enhances these processes [54]. Therefore, our results suggest that management practices altered the environmental conditions of the soils, which subsequently impacted the abundance of nutrient-cycling genes in the soils.

Many soil microbes act as biocontrol agents by producing and secreting bioactive substances (secondary metabolites) known as siderophores [55]. The production and secretion of siderophores are one of the various modes of biocontrol activity used by microbes to acquire the nutrient element, iron [56]. The analysis of microbial functional genes involved in plant growth promotion revealed higher abundances of the *pvd*D, *pvd*J, *pvd*I, and *mbt*H genes that are linked with the production and utilization of pyoverdine siderophore in the former grassland rhizosphere samples, thereby indicating that microbial communities in these soils might have high disease-suppressive capabilities. Our results revealed distinct dissimilarities in the diversity (ANOSIM, *p* = 0.01 and *R* = 0.52) of microbial functional genes responsible for plant growth promotion, as observed in the principal coordinate analysis (PCoA) (Figure 4) and the similarity percentage (SIMPER) analysis (Table 1). We attribute these dissimilarities to the different management practices involved in the fields, which have subsequently impacted the functional attributes of the soils. Although the α diversity for these genes was not significant between both fields, we observed higher α diversity indices in the F1 soils than in the F2 soils. Likewise, the PCoA plot displayed a close clustering of samples from the same field, with samples from a distinct field widely separated, pointing toward land-use and management differences (Figure 4). These results, in collaboration with the similarity percentage analysis (SIMPER) of the plant growth-promoting genes observed in the samples, are in agreement with our hypothesis that the diversity of microbial functional genes in F1 will differ from those of F2 due to land-use and management histories.

Microbial communities in soils contribute significantly towards soil organic carbon cycling and fixation, while changes and the pattern of changes in the soil organic carbon pool are regulated by the soil microbial community structure [57,58]. The impact of land-use and management histories on the distribution of carbon-cycling functional genes as assessed by our study revealed that specific gene families concerned with carbon cycling were preferentially associated with a particular soil. For example, the carbon fixation genes, *cbb*L (Ribulose bisphosphate carboxylase large chain), *cbb*Q (RuBisCo activation protein), *cbb*O (RuBisCo activation protein), and *rpe* (Ribulose-phosphate 3-epimerase), were statistically differentially abundant (α = 0.05) in the F1 rhizosphere (GZ) soils compared to *cod*H (carbon monoxide dehydrogenase large chain) and *cbb*X (probable RuBisCo-expression protein) that were statistically differentially abundant (α = 0.05) in the F2 rhizosphere (AG) soils (Figure 5b). Ribulose bisphosphate carboxylase (RuBisCo) is the principal enzyme in the Calvin–Benson cycle, which initiates the process of carbon fixation [28]. These results indicate that the agricultural management history of each field (grassland with no tilling and lower N fertilization, and continuous cultivation with tilling and higher N fertilization) might have provided similar conditions that favored broad colonization by microbial communities with the same functional groups in each field. Furthermore, the genes associated with methane degradation, *mxa*F, *fba*B, and *mmo*X, were significantly more abundant in the F1 rhizosphere soils than in the F2 rhizosphere soils. We suggest that higher and prolonged fertilizer use in the intensively cultivated soils (F2) may have influenced the lower abundance of the genes involved in methane degradation in these soils. Manoharan, Kushwaha, Ahrén, and Hedlund [1] also reported a higher abundance of genes in grassland soils than in cultivated soils. They further indicated that the continuous application of fertilizers in agricultural soils can inhibit methane breakdown in these soils. In our study, the higher abundance of genes involved in the degradation of methane in F1 soils is a confirmation of the results observed in the physicochemical components of the soils, which revealed higher OC, TC, and OM contents in the former grassland soils. These results, along with those of the microbial composition of the soils, confirm that the F1 soil environment is colonized by active methanotrophic microbial communities that metabolize and use methane as sources of energy and carbon, thereby helping to regulate methane flux in the environment.

The similarity percentage analysis (SIMPER) further revealed the extent of dissimilarity in the diversity of carbon-cycling genes between the GZ and AG soils, whereas the PCoA plot demonstrated that there were obvious differences in the abundance and distribution of carbon-cycling genes across the fields. Taken together, these results confirmed our second assumption in which we hypothesized that land-use and management histories would impact the diversity of the genes observed in the rhizosphere of the former grassland and the intensively cultivated land. Gaining insights into how the composition and diversity of microbial carbon-cycling genes in the rhizosphere are impacted by land use may further increase our knowledge of the effects of anthropogenic activities on carbon flux in the agricultural soil environment.

In our study, differences were observed in the chemical properties of both fields. The amounts of N-NO_3_, K, OM, TC, and OC were higher in the F1 soils than in the F2 soils, while N-NH_4_ content was higher in the F2 than in the F1 rhizosphere samples (Table S1). Land disturbances arising from long-term agricultural practices may impact the physicochemical properties of soils and alter the composition and properties of the soils’ biogeochemical interfaces [59]. Moreover, the soil microbial community structure and function may be impacted by land-use practices, resulting in an alteration of the soils’ chemical properties [8,60]. Long-term continuous agricultural practices, such as fertilization and tillage reduce soil quality and cause land degradation [61]. Based on our study, we suggest that the lower levels of OM, TC, OC, and N-NO_3_ observed in the F2 soils were due to intensive cultivation practices in the field, as continuous cultivation degrades the physicochemical properties of soils. These results coincide with those of Fujisao et al. [62], who indicated that continuous cultivation under conventional tilling reduces the contents of TC, TN, and exchangeable K in maize soils. The lower OC in F2 soils may also explain the reason for the lower OM in the soils. Long-term cultivation reduces OC, which contains about 58% of OM [63]. Therefore, land cultivation with heavy pieces of machinery and the persistent application of fertilizers over a long period increases the mineralization and degradation of OM and OC in soils [64]. Additionally, lower levels of soil organic matter (SOM) due to intensive cropping reduces soil fertility over time by depleting the stocks of important soil elements, such as N, P, and S [65]. This may be the case in our study, with the lowest levels of S observed in AG2 and completely absent in other F2 (AG1, AG3, and AG4) samples. Furthermore, conventional agricultural practices cause the acidification of soils. The literature has indicated that the extremely long use and heavy application of N fertilizer reduce soil pH [66,67]. As observed in our study, lower pH values were detected in F2 soils, indicating the influence of long-term intensive agriculture on the soils’ pH. This result is also consistent with the amounts of N-NO_3_ and N-NH_4_ found in the soils, as N in the form of nitrate (NO_3_) increases the pH in the rhizosphere, while N in ammonium (NH_4_) results in the acidification of the rhizosphere [68]. On the other hand, Sengupta et al. [69] reported lower pH in plow-till soils and higher pH in no-till soils, in line with the results of this study. Furthermore, the canonical correspondence analysis (CCA) showed that N-NO_3_, with a significance level of 0.05, contributed the most (31%) to the differences observed in the diversity and abundance of carbon-cycling and plant growth-promoting genes in the samples (Figure 7 and Table S4). Apart from N-NO_3_, our results also showed that other soil properties, including N-NH4, pH, OC, and OM, also contributed to the observed differences, as shown in the length of their vector arrows in Figure 7. These results agree with Li et al. [70], who reported that NO_3_, pH, NH_4_, and OC were influential factors that determined the abundance and distribution of functional genes in heavy metal-contaminated soils. The results of this study indicate that soil physicochemical properties also impact microbial functional gene composition and diversity in soils.

## 5. Conclusions

Shotgun metagenomic sequencing was applied on maize rhizosphere soils to elucidate the effects of land-use and management histories on the diversity and composition of microbial functional genes involved in plant growth promotion and carbon cycling. The differences in the abundance of functional genes involved in carbohydrate metabolism, carbon fixation, methane degradation, plant growth promotion, and nutrient mobilization are evidence that land-use and management histories impact microbial functions in agricultural soils. Moreover, among the soil properties, N-NO_3_ was the most influential in determining the composition and diversity of these genes across the samples; this indicates that soil chemical properties, which are also highly influenced by anthropogenic activities [59], are strong factors that affect microbial functions in soils. The taxonomic diversity revealed the abundance of microorganisms linked with important functions in the plant rhizosphere, with a majority more abundant in the former grassland rhizosphere; in addition, it reflected the effects of agricultural practices on the rhizosphere microbiota. The study increased our understanding of the relationship between plant health, biogeochemical nutrient cycling, the rhizosphere microbiome, and anthropogenic activities, all of which have direct or indirect effects on food production as the increasing human population exerts more pressure on crop yield. Future studies that target the genes expressed in these soils may help divulge the different microbial functional genes truly active in the rhizosphere soils and enhance our understanding of the functioning of microbial communities in the plant rhizosphere. In conclusion, this study confirmed that land-use history and management practices could impact the environmental conditions of soils, which may subsequently influence the ecosystem services (functions) of the microbial communities in the rhizosphere of agricultural soils.

## Figures and Tables

**Table 1:** Overall dissimilarities and the top shared plant growth-promoting and carbon-cycling genes with the most contribution to the dissimilarities between the samples.

Sample Pair	Ov. Avg. Dissimilarity	Contribution % of Plant Growth Promoting Genes			Ov. Avg. Dissimilarity	Contribution % of Carbon-Cycling Genes		
		*kup*	*ppk*	*trp*B		*cod*H	*lac*Z	*mxa*F
GZ and AG	28.00	8.70	7.67	5.76	26	12.43	11.93	10.4
GZ1 and GZ2	6.55	11.90	6.94	4.57	6.90	10.18	13.90	14.01
GZ1 and GZ3	18.08	9.87	6.05	3.60	15.96	6.72	13.35	7.40
GZ1 and GZ4	9.11	6.76	12.85	7.52	10.27	16.82	11.85	8.04
GZ2 and GZ3	12.81	8.24	5.20	2.91	10.83	3.96	11.29	2.85
GZ2 and GZ4	13.78	10.16	11.46	6.97	14.60	16.37	15.00	12.50
GZ3 and GZ4	24.20	9.80	8.67	5.12	24.15	10.70	13.64	8.08
AG1 and AG2	19.03	6.91	7.06	6.27	15.84	21.3	10.85	6.44
AG1 and AG3	48.0	6.83	8.29	7.02	45.58	19.82	12.86	4.93
AG1 and AG4	44.32	7.33	8.11	6.81	40.13	19.84	13.24	4.67
AG2 and AG3	31.93	6.74	9.47	7.74	32.71	18.18	14.05	3.65
AG2 and AG4	28.87	6.74	9.48	7.74	27.03	17.64	14.73	2.91
AG3 and AG4	6.00	1.72	9.00	8.53	7.12	18.40	8.21	7.16

Note: Ov. avg. stands for overall average; GZ represents all sites in field 1 (F1), including GZ1–GZ4; AG represents all sites in field 2 (F2), including AG1–AG4; GZ1–GZ4 represent each sample site in F1; AG1–AG4 represent each sample site in F2.

## Data Availability

The data files (reads in FASTQ format) were deposited at the NCBI SRA database under the BioProject accession No.PRJNA649682.

## Supplementary Materials

The following are available online at https://www.mdpi.com/article/10.3390/genes12091431/s1. Figure S1: Relative abundance of major microbial families found in the maize rhizospheric soils; Figure S2: Alpha diversity of genes involved in plant growth promotion in maize rhizospheric samples; Figure S3: Alpha diversity of genes involved in carbon cycling in maize rhizospheric samples; Table S1: Physicochemical parameters of soil samples; Table S2: Data of plant growth-promoting genes found in maize rhizospheric samples; Table S3: Data on of carbon-cycling genes found in maize rhizospheric samples; Table S4: The forward selection of physicochemical parameters that explains the most significant difference in the composition of the functional genes among the samples
