# Supplementary material for: Metagenomic Analyses of Plant Growth-Promoting and Carbon-Cycling Genes in Maize Rhizosphere Soils with Distinct Land-Use and Management Histories
Source: Genes (Basel). 2021 Sep 17;12(9):1431. doi: 10.3390/genes12091431 (PMC8466292; doi:10.3390/genes12091431)
Supplement: Supplementary file 1 [file genes-12-01431-s001.zip › genes-1358262-supplementary.pdf]

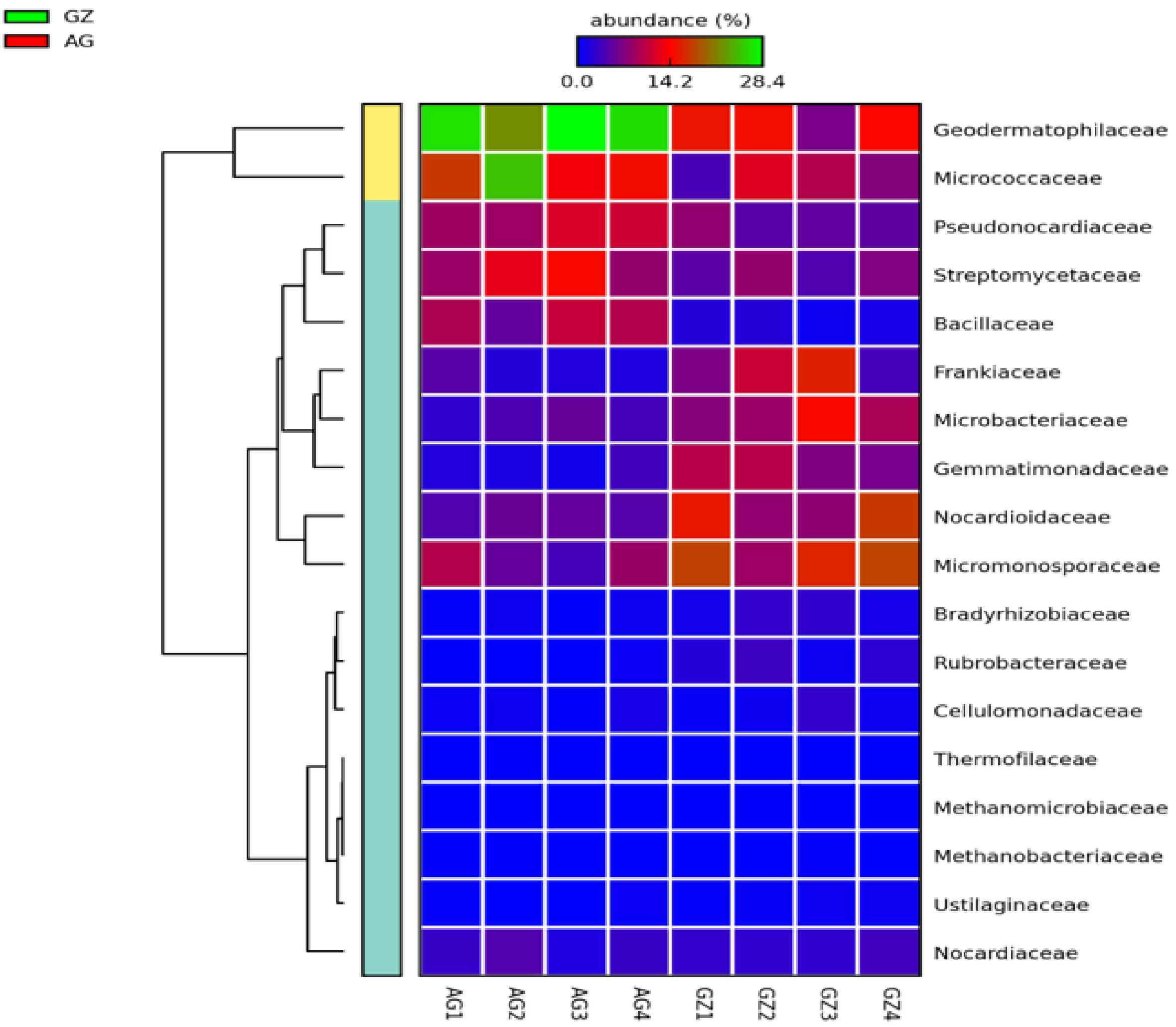

Figure S1: Relative abundance of major microbial families found in the maize rhizospheric soils

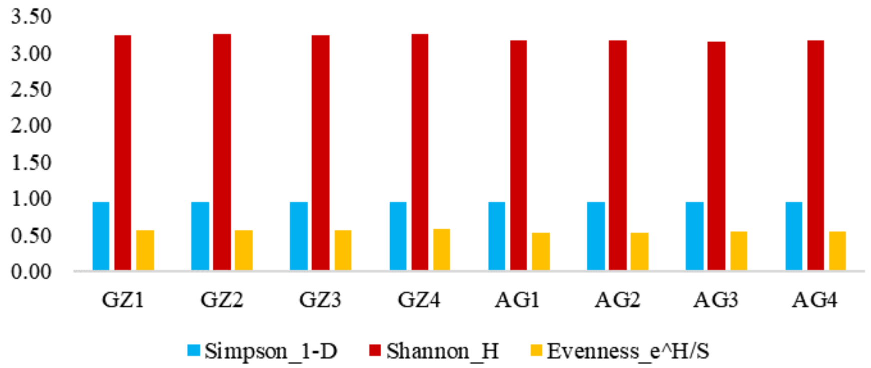

Figure S2: Alpha diversity of genes involved in plant growth promotion in maize rhizospheric samples

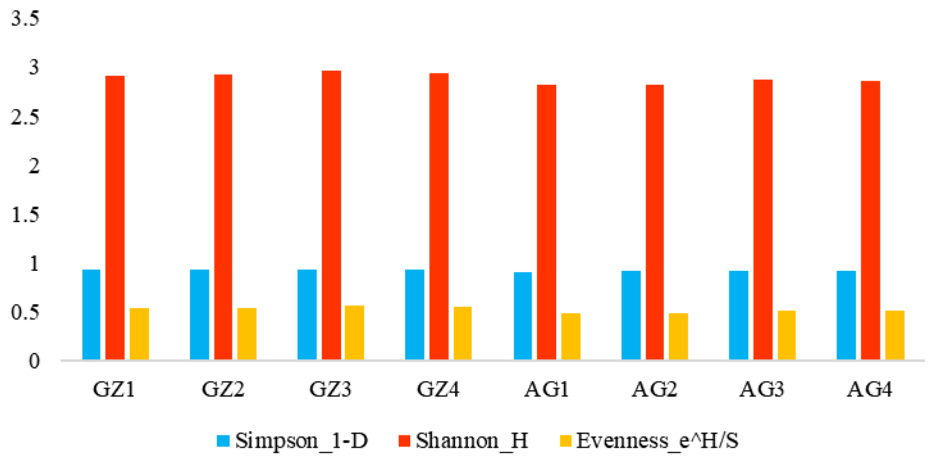

Figure S3: Alpha diversity of genes involved in carbon cycling in maize rhizospheric samples

Table S1: Physicochemical parameters of soil samples

Sample	Parameter	pH (H_2O)	S (mg/kg)	Total C (%)	P (mg/kg)	K (mg/kg)	N-NO3 (mg/kg)	N-NH4 (mg/kg)	Organic C (%)	Organic matter (%)
GZ1		6.79	16.2	1.24	24.29	338	16.56	3.22	1.04	4.02
GZ2		7.04	0.00	1.19	19.75	430	16.42	3.32	1.07	4.16
GZ3		6.64	2.78	1.18	40.39	338	18.02	3.68	1.06	4.19
GZ4		6.45	0.00	1.04	26.52	254	14.16	1.87	0.81	3.46
AG1		5.49	0.00	0.49	29.31	121	0.97	8.01	0.26	1.38
AG2		4.84	0.68	0.40	56.88	110	1.91	10.6	0.26	1.54
AG3		5.04	0.00	0.48	22.75	86.1	1.00	5.34	0.24	1.46
AG4		5.44	0.00	0.49	16.25	116	0.93	6.73	0.29	1.64

Table S2: Data of plant growth-promoting genes found in maize rhizospheric samples

Functional gene involved in	Gene code	GZ1	GZ2	GZ3	GZ4	AG1	AG2	AG3	AG4
2,3-butanediol and acetoin biosynthesis	*bud*B	411	496	508	358	805	600	227	285
	*bud*A	407	452	545	324	776	488	220	262
	*al*S	1661	1662	2255	1420	2529	1712	789	878
	*bud*C	1	5	6	0	3	5	0	3
4-hydroxybenzoate biosynthesis	*ubi*C	5	6	9	3	8	8	3	5
ACC deaminase activity	*acd*S	203	229	284	187	190	171	99	81
	*dcy*D	18	21	17	12	12	13	3	6
Ammonification	*ure*C	538	477	735	821	1023	607	516	792
Arsenate degradation	*gab*T	15	17	16	11	18	9	10	3
	*ars*C	505	555	618	402	543	365	226	211
Denitrification	*nir*K	25	29	50	47	37	14	7	21
	*nir*S	1590	1684	2374	1416	2138	1456	786	771
	*nor*B	20	15	20	15	8	7	5	0
Sulfur metabolism	*cys*C	954	1137	1329	1222	808	772	441	445
	*cys*I	419	515	783	385	410	277	157	160
	*cys*J	137	145	206	103	143	117	41	53
	*cys*H	355	367	465	304	449	383	172	211
	*cys*D	938	962	1275	770	1408	912	465	480
	*cys*N	814	743	1086	728	1429	955	499	482
IAA biosynthesis	*ipd*C	13	13	11	8	18	9	6	3
Nitrification	*amo*A	28	52	36	36	29	12	11	13
Nitrogen fixation	*nif*H	2	1	1	0	2	0	0	0
	*nif*A	366	540	481	222	179	94	36	77
	*fix*J	91	80	154	85	57	41	13	15
Oxidative stress	*sod*B	279	372	440	260	250	121	87	77
	GST	938	1205	1686	1001	888	543	280	331
	cat	1385	1523	2294	1215	2309	1598	805	813
Phenazine	*phz*F	523	495	646	384	661	463	243	265
Phosphorus cycling	*gdh*	759	898	1020	540	718	421	189	233
	*pp*X	752	824	1197	739	1105	762	420	410
	*pp*K	2036	2256	2646	1532	2370	1715	809	924
	*pst*A	794	905	1130	670	914	539	334	375
Potassium cycling	*Kef*A	103	131	172	91	127	76	42	44
	*Kef*B	353	474	626	289	283	163	68	102
	*K*up	1810	2187	2805	1545	2000	1359	715	693
	*Ktr*A	26	21	27	20	28	16	20	7
	*Ktr*B	44	73	58	46	48	19	10	12
Pyoverdine siderophore	*pvd*Q	25	13	21	17	21	18	2	9
	*pvd*L	122	183	248	125	108	75	32	29
	*pvd*D	190	224	296	143	154	87	37	52

	p*vd*I	101	128	132	91	77	63	25	26
	*Pvd*J	122	183	171	118	64	61	28	35
	*mbt*H	14	16	17	16	17	10	4	4
quorum sensing	*rib*B	672	786	947	553	805	503	237	299
Tryptophan biosynthesis	*trp*A	642	745	1063	606	1040	677	387	402
	*trp*B	1635	1780	1998	1340	2011	1429	689	798

Table S3: Data on of carbon-cycling genes found in maize rhizospheric samples

Functional gene involved in	Gene code	GZ1	GZ2	GZ3	GZ4	AG1	AG2	AG3	AG4
Metabolism of carbohydrate	*man*C	126	111	104	75	234	179	77	102
	*man*A	307	363	495	300	539	344	212	235
	*gal*K	560	614	825	419	899	572	279	361
	*glc*D	142	165	187	110	144	142	73	75
	*mel*A	674	605	837	547	700	679	368	390
	*lac*Z	2449	2834	3412	2023	2989	2297	1115	1224
Fixing of carbon	*gap*2	10	7	4	6	3	9	1	1
	*cod*H	2721	3003	3206	2116	4230	2871	1342	1586
	*cbb*L	139	97	285	150	70	70	36	35
	*cbb*R	7	10	12	2	14	8	4	3
	*cbb*O	17	19	20	18	13	5	3	2
	*cbb*Q	51	101	116	54	26	28	5	12
	*cbb*X	41	47	63	43	155	102	39	49
	*cbb*S	31	40	34	30	64	34	16	13
	rpe	736	997	1240	623	593	646	320	373
Degradation of hemicellulose	*ara*B	15	34	30	9	20	17	10	7
	*xyl*A	235	288	400	226	275	199	77	84
	*xyn*A	277	284	402	231	296	168	103	129
Degradation of methane	*glp*X	856	868	989	667	882	865	395	509
	*fba*B	689	851	998	505	424	358	202	267
	*mxa*F	1286	1674	1820	997	974	563	256	351
	*mmo*X	137	252	530	188	81	49	66	57
	*fba*A	552	654	846	759	603	254	285	353
Degradation of Starch	*glg*B	231	251	310	183	268	148	82	77
	*mal*Q	849	967	1245	806	1161	775	410	436
	*bgl*X	2056	1975	2794	1804	3402	2246	1108	1313
	*glg*C	1072	1178	1377	820	1301	891	487	468
	*abf*A	571	515	833	451	624	579	321	378
	*amy*A	812	908	1082	684	734	640	343	348
	*mal*Z	960	900	1072	791	964	956	494	509
	*tre*A	157	198	243	125	179	93	56	69
	*tre*C	70	49	68	41	88	43	22	27
Degradation of xenobiotics	*van*B	134	185	221	143	225	143	68	70
	*uid*A	57	56	81	53	97	40	24	29

Table S4: The forward selection of physicochemical parameters that explains the best difference in the functional genes composition among the samples

Physicochemical parameter	Contribution %	Pseudo-F	*p*-value
N-NO_3	31.3%	2.7	0.05
N-NH_4	20.4	2.1	0.07
pH	11.8	1.3	0.3
OM	12.2	1.5	0.25
OC	11.5	1.8	0.24
